# Investigation of Few-Layer Graphene–Ubiquitin Interactions with Optical Spectroscopy Techniques

**DOI:** 10.3390/nano15241873

**Published:** 2025-12-13

**Authors:** Burcu Gencay, Günnur Güler

**Affiliations:** Biophysics Laboratory, Physics Department, Izmir Institute of Technology, Urla, 35430 Izmir, Türkiye

**Keywords:** nanoparticle–protein interaction, protein secondary structure, optical spectroscopy, ubiquitin, few-layer graphene, FT-IR, CD, Raman, UV-Vis

## Abstract

Understanding the molecular mechanisms of protein–nanoparticle interactions is crucial for enabling the development of new applications in biomedicine and nanotechnology. Ubiquitin, an important and structurally small functional protein, plays a central role in numerous cellular processes. Therefore, in the current study, we focused on the few-layer graphene (FLG)–Ubiquitin complexes formed by exfoliating FLG structures using only water. Optical spectroscopic techniques (Raman, FT-IR, UV-Vis and circular dichroism) were employed to investigate these complexes on the molecular level. Overall, both CD and FT-IR data reveal that the formation of the FLG–Ubiquitin complexes occurred without inducing disordered structures in the protein. Based on the existence of a blue shift (hypsochromic shift) in the UV-Vis data, the presence of a single tyrosine and two phenylalanine residues in ubiquitin enables the detection of FLG-induced micro-environmental changes, particularly influencing the protein’s β-sheet and α-helix structures. The CD spectral results and CDPro quantitative estimations are in line with ATR FT-IR results, confirming the absence of disordered structure formation while altering the protein’s chirality. UV-Vis and CD spectroscopy results revealed concentration-dependent trends consistent with FLG–protein interactions that preserve the overall protein structure. This study has potential applications in both academic research and practical usage, particularly in biomedicine and nanotechnology specifically for FLG.

## 1. Introduction

Protein–nanoparticle interactions have been subject to scientific interest due to their possible biomedical applications [1,2,3,4]. The extremely small size of nanoparticles and their potential to penetrate nearly every part of the body necessitate their study in a wide range of areas, from drug delivery to emerging field of nanotoxicity [5,6,7]. Due to their exceptional electrochemical properties, high thermal conductivity, superior electrical conductivity and optical characteristics, graphene/graphene derivatives have found extensive applications in fields such as biomedicine and biosensor applications [8]. The interaction of proteins with graphene and graphene-based materials has emerged as a critical area of research. When proteins encounter graphene surfaces, they undergo adsorption driven by multiple non-covalent interactions, including hydrophobic Van der Waals forces, and electrostatic interactions [9,10]. These interactions can induce significant conformational changes in proteins’ secondary and/or tertiary structure, with consequences depending on the graphene’s form, oxidation level, and dimensions as well as the protein characteristics and environmental conditions [11,12].

Computational studies provided insights into the protein–graphene interactions, revealing that adsorption is driven by the different arrangements of hydrophobic and aromatic residues on their surfaces. In the presence of single-layer graphene systems, proteins often undergo extensive structural rearrangements, adopting parallel orientations relative to the graphene plane to maximize hydrophobic and π-π stacking interactions [13,14]. The choice of graphene derivatives can also influence this process. Few-layer graphene (FLG) enables simultaneous protein–surface contact and structural preservation (0.3–0.4 nm interlayer spacing prevents protein penetration between layers) while maintaining pristine domains suitable for future biosensor functionalization. Therefore, in our study, few-layer graphene (FLG) structures synthesized through the exfoliation method were exploited. When combined with biomolecules, FLG nanocomposites exhibit superior biocompatibility, enhanced biofunctionality, and low cytotoxicity, offering more advantages and greater development potential than traditional graphene-based materials in biological and biomedical fields [10]. Although they have been highly studied and widely recognized in the academic field, the fundamental interactions between nanomaterials and proteins remain not well understood [15,16]. A number of studies on nanoparticle–protein complexes have been conducted to gather relevant information by using various spectroscopic techniques such as circular dichroism (CD), FT-IR, UV/fluorescence spectroscopy, and others [17,18]. However, the interaction of few-layer graphene (FLG) and ubiquitin has not been studied so far.

Ubiquitin is a small signaling protein composed of 76 amino acids. Ubiquitin plays a crucial role in various cellular processes within living organisms, including proteasomal degradation, immune response, and DNA damage repair [19,20]. Due to its small size, low molecular weight, and favorable properties, ubiquitin not only serves as a model system for various biophysical and computational techniques but also acts as a target for studying interactions with nanomaterials [11,12,17]. Therefore, in the current study, we selected FLG:Ubiquitin complexes as a representative model to investigate the interaction between proteins and nanomaterials.

Various techniques have been employed for graphene synthesis; however, graphite exfoliation remains one of the most straightforward and widely utilized methods for producing graphene and graphene oxide. Graphene layers are stabilized by two distinct types of bonding interactions. Within each layer, carbon–carbon atoms are connected by strong covalent bonds with a bond length of approximately 0.142 nm. In contrast, adjacent graphene layers are held together by weak van der Waals forces, with an interlayer spacing of approximately 0.341 nm. This weak interlayer interaction facilitates the exfoliation of graphite into individual graphene layers [21]. Layered materials, such as graphite, are composed of two-dimensional platelets that are weakly stacked to form three-dimensional structures. Liquid-phase exfoliation (LPE) is a top-down approach for exfoliating natural graphite into few-layer graphene dispersed in liquid media. LPE has gained prominence due to its cost-effectiveness, scalability, and ability to produce defect-free graphene. Additionally, it allows for the deposition of dispersed graphene in various environments and on diverse substrates, which is not feasible with mechanical cleavage or growth methods [22,23,24].

Raman spectroscopy has thus far been the most used technique for determining the number of layers, N, in graphitic materials in a cost-effective way [25]. In our study, Raman spectroscopy as a vibrational spectroscopic technique was also chosen for the characterization of the structures obtained through exfoliation based on their molecular signatures. Fourier transform–infrared (FT-IR) spectroscopy offers a significant advantage in providing highly detailed insights into the structural and conformational changes in proteins [26,27,28]. Infrared spectroscopy is increasingly employed for protein and protein–nanoparticle analysis due to its ability to probe universally present bonds in peptides. Proteins generate nine characteristic IR absorption bands—amide A, B, and I−VII—of which the amide I and II bands are the most prominent vibrational features of the protein backbone. FT-IR spectroscopy allows structural analysis of proteins and peptides in diverse environments, with direct correlations between amide I band (1700–1600 cm^−1^) frequencies and secondary structure components. FT-IR second-derivative spectra are widely used to assess structural changes in nanoparticle–protein complexes [1,26,27,28].

Circular dichroism (CD) spectroscopy is a widely employed technique for investigating protein structures. Far-UV circular dichroism (CD) spectroscopy (180–250 nm) is primarily used in order to analyze the proteins. The characteristic secondary structures of the peptide backbone, including α-helices, β-sheets, turns, and disordered regions, significantly affect the CD spectrum. CD spectroscopy has been widely utilized for assessing protein folding, stability, intermolecular interactions, and ligand binding [27,29,30]. In this context, various algorithms have been developed and refined to facilitate the analysis of protein secondary structures [31,32]. UV-Vis spectroscopy is widely employed for investigating nanoparticle–protein complexes. However, it typically provides limited qualitative information regarding the binding of proteins to nanomaterials, primarily through the observation of peak shifts, broadening of the absorption spectra, and variations in spectral intensity [33,34]. To overcome these limitations, this study employs UV-Vis spectroscopy in conjunction with complementary spectroscopic techniques for a more comprehensive analysis [35,36].

In the current study, optical spectroscopic techniques, including Raman, FT-IR, UV-Vis and CD, were used to characterize exfoliated FLG and to analyze the effect of few-layer graphene (FLG) on the ubiquitin structure on the molecular level. FT-IR second-derivative analysis was utilized for tracking alterations in protein secondary structures and the CD results contributed to both secondary structure analysis and prediction in percentage, while UV absorption spectroscopy provided insights into the micro-environmental changes surrounding the single tyrosine (Tyr) and two phenylalanine (Phe) residues. Herein, the FLG–Ubiquitin complex was selected as a model system to explore protein–nanomaterial interactions.

## 2. Materials and Methods

### 2.1. Samples

Highly Oriented Pyrolytic Graphite (HOPG) nanostructures were kindly provided by Prof. Dr. Cem Çelebi, IZTECH-Physics Department. Ubiquitin from bovine erythrocytes was purchased from Sigma-Aldrich (St. Louis, MO, USA) and used without any further treatment. Physiologically suitable 20 mM potassium phosphate (KPi, pH 7.4) was used as protein solution buffer.

### 2.2. Ultrasonic Exfoliation of FLG in Water

An ultrasonicator (ISOLAB, D2012, 60W, Isolab Laborgeräte GmbH, Eschau, Germany) was used for the exfoliation method. The process was carried out for 2 h at a frequency of 40 kHz and a temperature of 313 K (±276 K), as described [37]. The temperature was monitored from the sonicator’s digital screen; when needed, a cold press was applied to sustain the temperature within the desired range. During this process, the temperature was regularly monitored. In order to keep the concentration at 6.7 mg/mL, water levels were cautiously monitored and as much water that had evaporated was provided back. After the process was completed, the solution was centrifuged at 1500 relative centrifugal force for 30 min.

### 2.3. Confocal Raman Microspectroscopy

For the characterization of HOPG (in flake form) and FLG (exfoliated and sonicated), a Renishaw inVia Qontor Raman confocal spectrometer (Renishaw plc, Wotton-under-Edge, UK) was used combined with the Renishaw Centrus 4AUL44-1040 × 256 detector equipped with a 532 nm laser, used in normal mode. A grating of 2400 lines/mm was preferred for the visible region. Exposure time was set to 10 s; laser power (50 mW) was used as 100%. Raman data were recorded in the spectral region between 120 cm^−1^ and 3500 cm^−1^ on a gold substrate. Data visualization and analysis were performed using OPUS 8.7 (Bruker, Ettlingen, Germany) and OriginPro, Version 2025 (OriginLab Corporation, Northampton, MA, USA).

### 2.4. Attenuated Total Reflection Fourier Transform–Infrared Spectroscopy (ATR FT-IR)

Samples were prepared by mixing the FLG solutions with protein solutions at FLG:Ubiquitin ratios of 0:1 (refers to a blank ubiquitin solution), 1:1 (the FLG:Ubiquitin complex) and 1:0 (blank FLG solution). All the samples were shaken at 310 K at 300 rpm for 2 h. Measurements were performed by using an FT-IR spectrometer (Perkin Elmer, UATR Two, PerkinElmer, Inc., Shelton, CT, USA) coupled with an MIR-TGS detector and an attenuated total reflection (ATR) unit. The FT-IR spectra were recorded at room temperature in the spectral range of 4000–600 cm^−1^ (mid-IR) under equal conditions for the samples of buffer solution, FLG solution, protein solution and FLG–Ubiquitin complex. The spectrum of air was recorded when the ATR unit (diamond, single reflection) was empty and clean. A total of 128 scans were averaged before Fourier transformation, taking the air spectrum as background. The resolution was 4 cm^−1^ and the data interval was 1 cm^−1^ during the measurements. The second derivative of the spectra was computed using the Savitzky–Golay algorithm with 9 smoothing points to resolve the superimposed bands. The second derivative of the spectra was calculated by using OPUS 8.7 (Bruker, Germany), visualized by OriginPro, Version 2025 (OriginLab Corporation, Northampton, MA, USA).

### 2.5. UV-Vis Spectroscopy

The samples were adjusted to the FLG/Ubiquitin ratios of 1:0, 0:1, 1:1 and 4:1 with a constant protein concentration of 0.15 mg/mL. All samples were shaken at 310 K and 300 rpm for 2 h. Measurements were performed using the Perkin Elmer LAMBDATM 950 UV/Vis/NIR spectrophotometer (PerkinElmer, Inc., Shelton, CT, USA) at room temperature. Spectra were obtained in the range of 200–800 nm. Each of the samples was placed in 0.7 mL quartz cuvettes with a pathlength of 10 mm. The acquisition step was set to 1 nm. Corresponding deionized water and buffer mixtures were taken as background. Data visualization and analysis were also performed using OPUS 8.7 (Bruker, Germany) and OriginPro, Version 2025 (OriginLab Corporation, Northampton, MA, USA).

### 2.6. Circular Dichroism (CD) Spectroscopy

A CD spectrometer Jasco J-1500 (Jasco, Tokyo, Japan) was used for CD measurements. The FLG/Ubiquitin complexes were prepared (0:1, 1:1, 4:1, 1:0) by adding various amounts of FLG to a constant protein concentration of 0.15 mg/mL. Each sample was shaken at 310 K at 300 rpm for 2 h; the samples were then placed in quartz cuvettes (volume: 400 μL; pathlength: 0.2 cm) and measured at room temperature. CD spectra were recorded in the range of 190–350 nm with the parameters as follows: bandwidth: 1.0 nm, data pitch: 0.1 nm, DIT: 4 s, number of accumulation scans: 8 and scanning speed: 50 nm/min. CD is reported in degrees of ellipticity (θ), which is defined as the ratio of the minor to the major axis of the ellipse formed by the electric field vector of elliptically polarized light after its transmission through a chiral sample, upon which linearly polarized light was initially incident [32].

The protein secondary structure analysis from the CD spectra was accomplished by a curve fitting program in CDPro software (version 4.31) using the CONTINLL method [27]. According to this method, the analysis of a CD spectrum identifies six secondary structural elements: regular and distorted α-helixes, regular and distorted β-sheets, turns, and unordered structures [38]. For simplicity, the percentage values of regular and distorted α-helixes were summed to determine the total α-helix content, while the values of regular and distorted β-sheets were combined to obtain the total β-sheet content. Data visualization and analysis were also performed using OPUS 8.7 (Bruker, Germany) and OriginPro, Version 2025 (OriginLab Corporation, Northampton, MA, USA).

## 3. Results and Discussion

### 3.1. Characterization of HOPG and FLG

#### 3.1.1. Raman Spectroscopy Reveals the Structural Integrity of Few-Layer Graphene Structures

Raman spectroscopy is a predominant technique for the structural characterization of graphite, graphene, and nanostructured carbon-based materials [25,39]. In graphitic materials the D band arises from disordered carbon atoms, while the G band corresponds to the in-plane vibration of sp^2^ carbon atoms and can be utilized to assess the thickness of multilayer graphene. Accordingly, the presence of a G band arising around 1580 cm^−1^ and a 2D band positioned around 2700 cm^−1^ is a valid characteristic of graphene [40], and were observed clearly before and after the exfoliation process. The I_G/I_2D ratio and width of the 2D peak detected around 2700 cm^−1^ have valuable information regarding the number of layers of graphene material [41].

**Figure 1:**
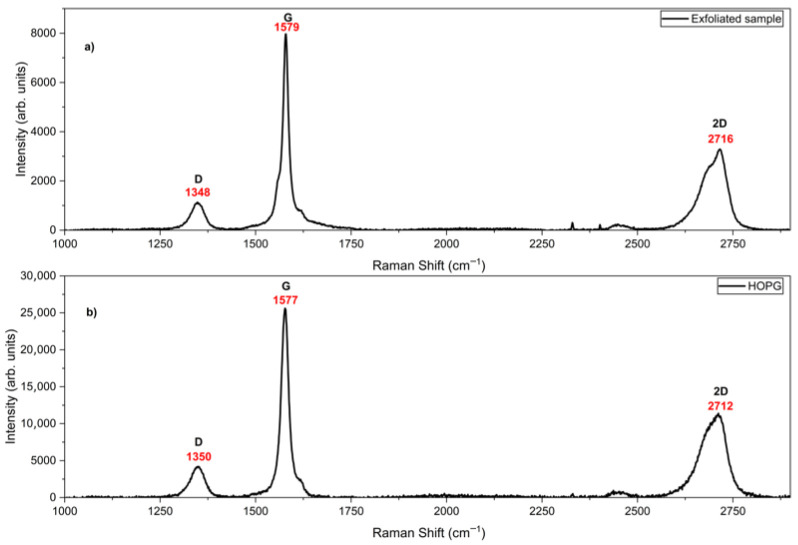
The Raman spectra of the (**a**) exfoliated samples and (**b**) HOPG.

The Raman spectrum of HOPG (Figure 1b) exhibits peaks at 1350 cm^−1^, 1577 cm^−1^ and 2712 cm^−1^ which correspond to the D band, G band and 2D band, respectively, while the Raman spectrum of exfoliated samples (Figure 1a) shows peaks at 1348 cm^−1^, 1579 cm^−1^ and 2716 cm^−1^, corresponding to the D band, G band and 2D band, respectively. The position of the 2D peak was shifted from 2712 cm^−1^ to 2716 cm^−1^ after the exfoliation process. The I_G/I_2D ratios of HOPG and exfoliates sample were calculated as 2.2 and 2.4, respectively. The FWHM of the 2D band of the exfoliated sample was approximately 74 cm^−1^. It was formerly reported that an I_G/I_2D >1.3 and an FWHM of the 2D band higher than 60 cm^−1^ indicate the presence of few-layer graphene [42]. In light of these results, the exfoliated samples probed here have few-layer graphene structures. The weak D peaks observed at 1350 cm^−1^ (Figure 1b) and 1348 cm^−1^ (Figure 1a) in the samples indicate high crystallinity. The D peak signal (generally expected around 1350 cm^−1^) exhibited no substantial variation in the exfoliated sample, which demonstrates that no defect was generated during the sample preparation process.

#### 3.1.2. UV-Vis Absorption Analysis Confirming Non-Oxidized FLG Formation

**Figure 2:**
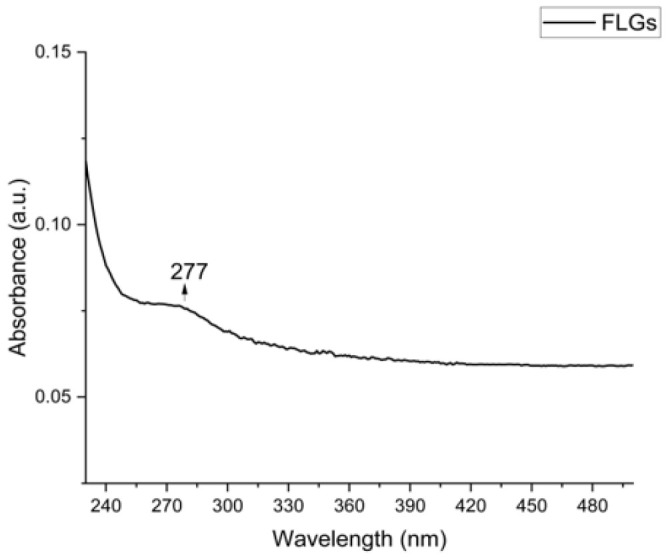
The UV-Vis absorption spectrum of exfoliated sample, FLG.

UV-Vis measurements were performed to determine whether the exfoliated samples had undergone oxidation, which can be detected by a peak around 230 nm [43]. It is known from UV-Vis spectroscopic studies that the characteristic spectrum of graphene/graphene materials exhibits a prominent peak around 270 nm. In our results (Figure 2) we have detected the mentioned peak at 277 nm, indicating the presence of graphene. In conjunction with this result, the exfoliation process was found to be consistent with the Raman results, indicating that it did not cause any damage to the material’s characteristic structure. Additionally, no peak in the neighborhood of 230 nm, associated with the presence of graphene oxide, was observed. This suggests that the FLG obtained was not oxidized to a detectable extent according to the UV-Vis spectra [36,44]. Multiple repeated measurements performed on FLG yielded consistent results, in line with each other.

### 3.2. The FLG–Ubiquitin Interactions

#### 3.2.1. FT-IR Spectroscopic Analysis Reveals FLG-Induced Conformational Changes in Ubiquitin

**Figure 3:**
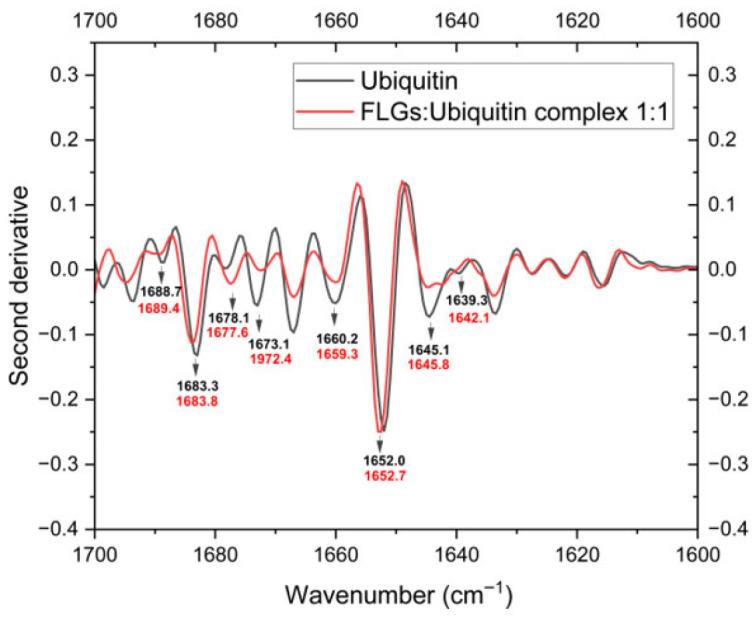
The FT-IR second-derivative spectra of amide I band for the ubiquitin protein (black solid line) and for the FLG:Ubiquitin complex (the 1:1 ratio, red solid line).

The FT-IR technique is a valuable tool for structural analysis and monitoring changes in the secondary structure of proteins and/or polypeptides [27,30,45,46]. A significant advantage of FT-IR spectroscopy for structural characterization is that it is not constrained by the protein size or the physical state of the samples. In the current study, FT-IR was used to monitor the conformational changes in ubiquitin’s secondary structure due to the presence of FLG. In the FT-IR second-derivative spectrum, the amide I region (Figure 3) shows that the IR peak absorbing at 1652 cm^−1^ was upshifted towards 1653 cm^−1^, indicating that FLG causes a slight conformational change in the protein’s alpha-helical structures. Given that there is no significant change in the spectral pattern in the 1625–1600 cm^−1^ region (due to absorption of amino acid side chains such as Lys, Asn and Trp [46]), the spectral change detected at 1652 cm^−1^ (due to α-helix) is reasonable, indicating the local interactions of ubiquitin with FLG. Such small shifts (on the order of a few wavenumbers) are expected for regular secondary structural elements as they produce highly collective and stable vibrational modes. However, large shifts (greater than 5–10 cm^−1^) accompanied by band broadening are typically observed when the protein is completely unfolded (denatured) [27]. Based on our data, the IR signals absorbing in the 1625–1640 cm^−1^ and 1670–1690 cm^−1^ spectral regions (due to β-sheets and turns) also exhibit slight shifts in the presence of FLG while the IR signals arising from the amino acid side chains in the 1625–1600 cm^−1^ region overlap without discernible shifts. By comparing the IR second-derivative spectra of blank ubiquitin and the 1:1 complex, we can conclude that the presence of FLG might have induced minor changes in the secondary structures of the protein without causing unordered patterns. These changes strongly reveal that FLG can affect a protein’s secondary structure in a versatile manner [for band assignments, see refs. [27,28,46]].

#### 3.2.2. CD Analysis Unveils FLG-Induced Conformational and Chirality Changes in Ubiquitin

The far-UV region of CD spectra, between 250 and 190 nm, has been widely used to analyze the secondary structures of proteins, including α-helixes, β-sheets, and random coils. It is a simple, fast, and non-destructive method for estimating the conformations of proteins. The CD spectra of the α-helix conformation typically show strong negative bands at 208 nm (π → π*) and 222 nm (n → π*) with a positive peak around 190 nm (π → π*). The β-sheet structures exhibit a negative band around 215 nm (n → π*) and a positive band around 195 nm (π → π*). However, the experimental results of β-sheet conformations may show significant variation due to structural differences, such as parallel and antiparallel arrangements, β-turns, and twists. Random coil conformations are also detectable in CD spectra by a relatively weak positive band at approximately 212 nm (n → π*) and a negative band around 195 nm (π → π*) [32,32,36,47].

**Figure 4:**
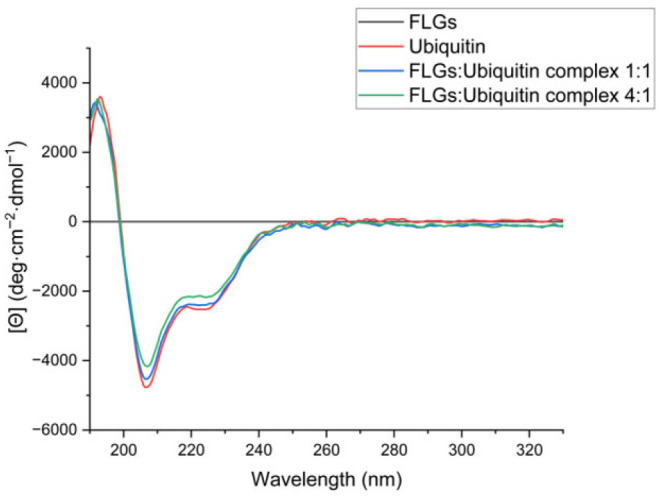
The CD spectra of FLG (black), ubiquitin (red) and the FLG:Ubiquitin complexes at the ratios of 1:1 (blue) and 4:1 (green).

In our results (Figure 4), the spectrum of ubiquitin shows two distinct minima at approximately 207 nm and 222 nm and a positive band around 195 nm. These minima are characteristic signals corresponding to the alpha-helix structures in the secondary structure of the protein. Observation of the CD signal at 217 nm corresponds to beta-sheets in the secondary structure of the protein [30,36]. When the CD spectra of the FLG:Ubiquitin complexes (ratios of 1:1 and 4:1) were examined, concentration-dependent spectral changes were clearly detected. The molar ellipticity values gradually decreased, indicating a change in chirality of the protein in the presence of FLG. Minimal changes appeared at the 1:1 ratio, while at the 4:1 ratio, the intensities of the 208 nm and 222 nm bands decreased noticeably. CD spectra of FLG:Ubiquitin complexes at both ratios of 1:1 and 4:1 showed progressive decreases in molar ellipticity compared to neat ubiquitin, which indicates that FLG induces concentration-dependent conformational changes in proteins without causing disorder. Despite these changes, the overall spectral profile remained characteristic of folded ubiquitin, indicating that chirality was altered without inducing protein unfolding. These findings altogether strongly indicate that FLG interacts with the ubiquitin protein and induces conformational rearrangements without causing a disordered structure in the secondary structure of the protein. There was no CD signal detected from FLG due to their intrinsically non-chiral nature.

The assignments of the secondary structure elements were obtained by a software program, CDPro, using the CONTINLL method. This method uses the reference sets of proteins, whose 3D structures are well known from X-ray diffraction studies. A 42-protein reference set of those databases was used for each CD spectrum between 190 and 240 nm. Our CD data estimates that ubiquitin consists of 3.9% alpha-helixes, 39.8% beta-sheets, 19.8% turns and 36.5% unordered structures; the complex with the 1:1 ratio includes 3.8% alpha-helixes, 39.8% beta-sheets, 19.8% turns and 35.6% unordered structures; and the complex with the 4:1 ratio gives 3.7% alpha-helixes, 40.4% beta-sheets, 19.7% turns and 36.1% unordered structures. Estimates showed minimal variation between native structures and complexes (≤0.6% change in any structural elements). This percentage is not statistically significant for a 76-residue protein, where each residue represents ~1.3% of the sequence. Nevertheless, the concentration-dependent ellipticity reduction combined with the preserved overall spectral characteristics suggests progressive interaction. The presence of FLG resulted in a notable change around 217 nm in the CD spectrum of ubiquitin (Figure 4), indicative of a reduction in the protein’s β-sheet content [13], agreeing with our spectral results. The secondary structure predictions were compared with the XRD results reported in the literature. Estimations for β-sheet structures exhibit a high degree of agreement [48]. It could be interpreted that the structural elements calculated by the method as ‘unordered structures’ correspond to alpha-helix structures. The results of the CD measurements and secondary structure estimations indicate that the interaction of ubiquitin with FLG causes alterations in the protein structure while influencing overall chirality without leading to the disruption of secondary structural elements.

#### 3.2.3. UV-Vis Spectroscopic Characterization of FLG-Induced Aromatic Micro-Environmental Changes in Ubiquitin

**Figure 5:**
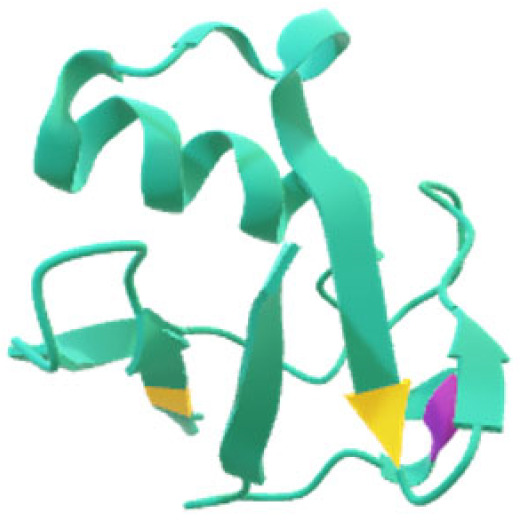
The 3D structure of ubiquitin showing Tyr (purple) and Phe (yellow) residues in color codes (reproduced in the PDB web from PDB ID: 2ZCC).

**Figure 6:**
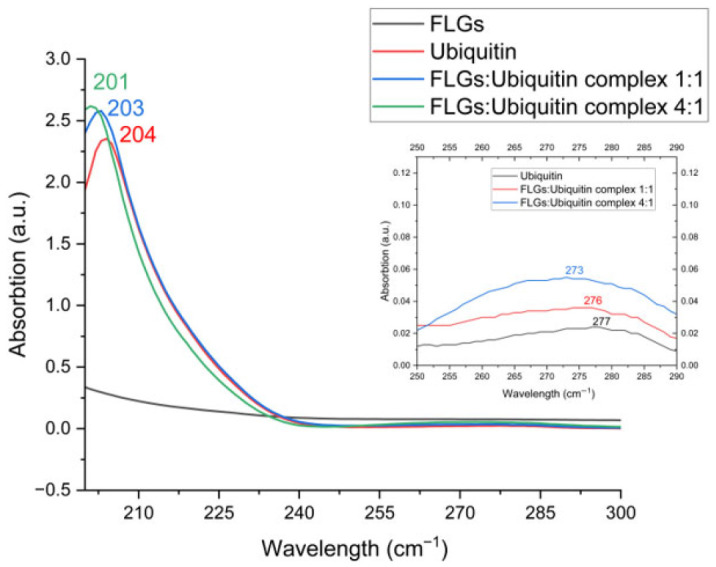
The UV-Vis spectra for FLG (black), ubiquitin (red), the 1:1 complex (blue) and the 4:1 complex (green).

The UV absorption of proteins between 180 and 230 nm primarily arises from π → π* transitions in peptide bonds. In the 230–300 nm range, absorption is mainly influenced by the aromatic side chains of tryptophan (Trp), tyrosine (Tyr), and phenylalanine (Phe) residues, with a minor contribution from disulfide bonds near 260 nm [49]. In this study, ubiquitin, used as a model protein, contains only one tyrosine (Tyr) and two phenylalanine (Phe) residues. The Phe residues are located on the β-sheet structures of the protein, while the Tyr residue is positioned on the α-helix structure (Figure 5). The UV-Vis spectrum of ubiquitin exhibits signals in the 200–250 nm range (Figure 6), which provides insights into the protein’s secondary structure. This spectral range provides information regarding the secondary structure of the protein [50,50,51].

The UV-Vis absorption spectra for the 0:1, 1:1 and 4:1 complexes showed two prominent peaks. Signals originating from secondary structure elements are observed in the 201–204 nm range (203 nm for the 1:1 complex), while a broad signal in the 273–277 nm range (276 nm for 1:1 complex) is attributed to phenylalanine (Phe) and tyrosine (Tyr) residues. The spectra indicate that the spectral properties of these amino acids contribute to the absorption bands within the structure of ubiquitin. In contrast, the spectrum of FLG presents a significantly lower signal. Upon examining the FLG:Ubiquitin complexes, a blue shift (hypsochromic shift [52]) was clearly observed. The related blue shift arising from alterations in the secondary structure of the protein induced by FLG is due to the π-π stacking between aromatic residues of the protein and the backbone of graphene [52]. The spectral shift occurring approximately from 277 nm to 273 nm shows changes in the microenvironment of phenylalanine (Phe) and tyrosine (Tyr) residues. These shifts demonstrated that FLG interacts with the ubiquitin protein, causing conformational and micro-environmental changes in ubiquitin’s structure. Another rational insight comes from the progressive hypsochromic (blue) shift with increasing FLG concentration (native: 277 nm; 1:1 complex: 276 nm; 4:1 complex: 273 nm), representing a total shift of 4 nm from native to the highest FLG concentration. Considering the positions of the tyrosine (Tyr) and phenylalanine (Phe) residues within the protein’s structure (Figure 5), the overall spectral trend highlights that FLG interacts with ubiquitin, inducing micro-environmental changes around Phe and Tyr residues. It is evident that changes occur in the vicinity of both the β-sheet and α-helix structures. The absence of tryptophan in the protein’s structure, along with the presence of only three aromatic residues (one tyrosine and two phenylalanine) in total, contributes to the low intensity and broad band profile.

In the current study, the ultrasonication process, performed using an ultrasonic bath, was preferred as a rapid, simple, and clean method to produce few-layer graphene (FLG) and yielded successful results. Raman analyses demonstrated that the exfoliated graphene structures remained intact, with no structural degradation, and that FLG was successfully formed. The data obtained from the second derivative analysis of the ATR FT-IR spectrum clearly demonstrated that the presence of FLG induces measurable changes in the alpha-helix, beta-sheet, and turn structures that constitute the secondary structure of the protein. The absence of disordered structure formation is a significant finding for the future of this study. The CD results are in agreement with the findings from ATR FT-IR analyses. This further confirms the absence of disordered structure formation while revealing a reduction in beta-sheet components and changes in the chirality of the protein. UV-Vis measurements are consistent with results obtained from ATR FT-IR and CD analysis. The results indicate that FLG:Ubiquitin complexes form without leading to structural disorder and that the presence of FLG induces minor changes in the secondary structures of the protein, as supported by the progressive spectral profile in FLG concentration CDPro results. Since the ubiquitin protein contains only one tyrosine (Tyr) and two phenylalanine (Phe) residues, the UV-Vis results provide insights into the micro-environmental changes surrounding these amino acids. Spectroscopic data clearly reveal that the micro-environments of these side chains are affected by the presence of FLG.

The studies by Mondal et al. [12] and Malik et al. [11] have been incorporated, both of which demonstrate graphene oxide–ubiquitin interactions and conclude that the protein’s structural integrity was largely preserved. Their findings are in line with our observation that FLG induces minor structural changes without causing disorder. This consistency across FLG (our study), single-layer graphene [13,14], and graphene oxide systems [11,12] establishes that the degree of structural perturbation depends critically on material properties such as the layer number, oxidation state, and surface chemistry. Wei’s team investigated ubiquitin folding upon absorption to a graphene surface using discontinuous molecular dynamics [14]. According to their study, after the first contact, the alpha-helix part rolled down towards graphene surface. The beta-sheet content started to collapse onto the surface while aligning parallel to the surface. After that, beta-sheets became random coils. The first contact was made by Leu50 due to hydrophobic interactions, followed by Leu56 and Ile61. Tyr59 and His68 residues were responsible for π-π interactions. Compared with our experimental design, the key difference lies in the structural constraints implied by our FLG structure. In Wei’s work, ubiquitin can lie parallel to the graphene, which leads to structural collapse. For an FLG structure the interlayer spacing is approximately 0.3–0.4 nm. As a result of this, ubiquitin cannot penetrate the graphene layers. Therefore, it cannot align in a parallel manner, which prevents further interactions and hence complete disorder. This geometric constraint may explain why beta-sheet reduction is observed. In FLG, these π-π interactions of course are highly likely to occur at both the top and bottom surfaces of the multilayer structure. It also creates multiple interaction sites which roll down towards the graphene surface. This ensemble point of view shows controlled perturbations rather than complete denaturation. The observed minor FT-IR and UV shifts and the CD results are consistent with our hypothesis of localized conformational alterations rather than overall protein unfolding.

## 4. Conclusions

The present work reveals that few-layer graphene alters the secondary structure of ubiquitin in a controlled manner, maintaining its overall conformational stability. Our findings demonstrate that FLG interacts with ubiquitin without inducing structural disorder while leading to minor alterations in the regular protein secondary structures and changes in protein chirality. The FT-IR second-derivative analysis confirmed alterations in the secondary structure, while CD spectroscopy provided additional insights into structural modifications upon FLG concentration and chirality shifts. Notably, the UV-Vis results support the CD findings by corroborating the observed changes in β-sheet structures. Furthermore, UV-Vis absorbance spectroscopy enabled an evaluation of the micro-environmental changes surrounding the single tyrosine and two phenylalanine residues, highlighting their sensitivity to FLG interactions via a concentration-dependent spectral trend.

The presence of multiple layers in the FLG structures enabled the formation of a different complex to those reported in the existing literature, as the protein could not penetrate between the layers. The interlayer regions remained pristine, and they are highly amenable to modification for sensor technologies. Moreover, the existence of these layers may provide an important alternative for sensor-based applications as they allow the protein to interact with the graphene surface without being fully adsorbed onto it and losing its structure.

These findings provide a clear understanding of how few-layer graphene can modulate protein conformation while preserving overall structural integrity, offering valuable guidance for designing protein–nanomaterial interfaces in biomedical and nanotechnological applications. Further work is in progress to analyze thermodynamic stability and perform detailed mapping of conformational transitions and energy transfer pathways in the FLG:Ubiquitin complexes by using FT-IR and CD spectroscopies.

## Data Availability

Data that support the findings of this study are available within the article and from the corresponding author upon request.

## Abbreviations

The following abbreviations are used in this manuscript:

FLG	Few-layer graphene
ATR FT-IR	Attenuated total reflection Fourier transform–infrared spectroscopy
CD	Circular dichroism
LPE	Liquid-phase exfoliation
UV-Vis	UV-Vis, ultraviolet–visible
